# Variabilité inter et intra-opérateur de l’analyse des paramètres spermatiques: résultat d’un programme de contrôle de qualité

**DOI:** 10.11604/pamj.2016.25.115.9158

**Published:** 2016-10-26

**Authors:** Salima Daoud, Nozha Chakroun-Feki, Afifa Sellami, Leila Ammar-Keskes, Tarek Rebai

**Affiliations:** 1 Laboratoire d’Histologie-Embryologie et Biologie de la Reproduction, Faculté de Médecine de Sfax, Université de Sfax, Tunisie

**Keywords:** Contrôle interne de qualité, spermogramme, mobilité, concentration, morphologie, Internal quality control, semen analysis, motility, concentration, morphology

## Abstract

**Introduction:**

L’analyse du sperme est d’une importance majeure dans l’exploration de l'infertilité masculine. Afin de s’assurer de la fiabilité des résultats rendus, l’implantation du management de qualité en spermiologie est devenue une nécessité.Le but de ce travail a été d’évaluer la variabilité intra- et inter-opérateurau cours de l’analyse des paramètres spermatiques au sein de notre laboratoire de spermiologie, à travers la mise en place d’un programme de contrôle de qualité.

**Méthodes:**

Quatre opérateurs ayant des niveaux d’expérience différents ont participé à l’étude. La variabilité inter-individuelle des résultats des lectures de la mobilité, la concentration et la morphologie spermatique a été évaluée sur plusieurs échantillons de sperme de qualités différentes. Pour chaque paramètre spermatique, la variabilité intra-individuelle a été évaluée en analysant les résultats des lectures de plusieurs aliquotes issus de chacun des échantillons utilisés.

**Résultats:**

Les coefficients de variation moyens inter-opérateurs ont été de12.8%, 19.8% et 48.9% pour la mobilité, la concentration et la morphologie spermatique, respectivement. Les coefficients de variation moyens intra-opérateurs ont été de6.9%, 12.3%et 42.7% pour la mobilité, la concentration, et la morphologie spermatique, respectivement. Mis à part quelques écarts (erreurs aléatoires), la plupart des mesures réalisées ont été dans les limites d’acceptabilité pour l’ensemble des opérateurs.La variabilité de l’évaluation morphologique des spermatozoïdes a été particulièrement influencée par le niveau d’expérience de l’opérateur.

**Conclusion:**

Les résultats de cette étude mettent l’accent sur la nécessité d’une formation adéquate du personnel de laboratoire, et de la participation régulière aux contrôles de qualité internes afin de minimiser les divergences et d’améliorer la fiabilité des résultats.

## Abstract (en)

**Introduction:**

Semen analysis is a key part of male infertility investigation. The necessity of quality management implementation in the andrology laboratory has been recognized in order to ensure the reliability of its results. The aim of this study was to evaluate intra- and inter-individual variability in the assessment of semen parameters in our laboratory through a quality control programme.

**Methods:**

Four participants from the laboratory with different experience levels have participated in this study. Semen samples of varying quality were assessed for sperm motility, concentration and morphology and the results were used to evaluate inter-participant variability. In addition, replicates of each semen sample were analyzed to determine intra-individual variability for semen parameters analysis.

**Results:**

The average values of inter-participant coefficients of variation for sperm motility, concentration and morphology were 12.8%, 19.8% and 48.9% respectively. The mean intra-participant coefficients of variation were, respectively, 6.9%, 12.3% and 42.7% for sperm motility, concentration and morphology. Despite some random errors of under- or overestimation, the overall results remained within the limits of acceptability for all participants. Sperm morphology assessment was particularly influenced by the participant’s level of experience.

**Conclusion:**

The present data emphasize the need for appropriate training of the laboratory staff and for regular participation in internal quality control programmes in order to improve the reliability of laboratory results.

## Introduction

Le spermogramme est une étape clé dans l’exploration d’une infertilité masculine. Souvent demandé de première intention, il consiste à analyser les paramètres spermatiques de base: volume et pH de l’éjaculat, concentration, mobilité, vitalité et morphologie des spermatozoïdes. La réalisation du spermogramme est basée sur l’évaluation microscopique d’un observateur humain, d’où un risque accru d’erreur comparativement à d’autres analyses biologiques effectuées selon des procédures automatisées [1]. La variabilité des résultats est étroitement liée à la qualité de l’apprentissage initial, au niveau de compétence de l’examinateur et au choix de la méthode d’analyse par le laboratoire [1]. Malgré les essais de standardisation des procédures d’analyse du sperme notamment par la publication régulière par l’Organisation mondiale de la Santé (OMS) de recommandations de bonnes pratiques en spermiologie [2–4], une hétérogénéité des pratiques et des résultats a été soulignée par plusieurs rapports [5–7]. Ceci ne peut être sans conséquences pour les couples infertiles, compte tenu de l’importance des résultats du spermogramme dans leur prise en charge aussi bien diagnostique que thérapeutique.

La nécessité du contrôle de qualité (CQ) en biologie de la reproduction a été reconnue depuis la fin des années 80 [8, 9]. Il a fallu cependant attendre l’apparition de la 5ème édition du manuel de l’OMS [4] pour que des recommandations relatives à ce sujet soient émises. Par définition, le contrôle interne de qualité (CQI) est l’ensemble des mesures permettant à un laboratoire de vérifier la fiabilité et l’exactitude de ses résultats.Il permet de détecter et d’identifier les erreurs, permettant ainsi de prendre des mesures correctives adaptées. Le contrôle de qualité inter-laboratoire ou contrôle externe de qualité (CQE) permet de déterminer les performances d’un laboratoire en les comparant avec celles d’autres laboratoires. Malgré les encouragements, le taux de participation des laboratoires d’andrologie à des programmes de contrôles de qualité internes et externes reste faible [5, 6, 10]. Le manque de législation en la matière dans plusieurs pays pourrait en être une explication. Il est cependant à noter que le caractère complexe et chronophage des procédures de contrôle de qualité, ainsi que l’absence de prérequis et de formation en assurance qualité pour la plupart des biologistes de la reproduction, pourraient constituer un frein à la généralisation de l’application de ces mesures.

L’objectif de cette étude a été de déterminer le niveau de performance d’un groupe d’opérateurs ayant des niveaux d’expérience différents en spermiologie, par le biais d’un protocole de contrôle interne de qualité mis en place dans notre laboratoire.

## Méthodes

### Les participants

Quatre personnes ont participé à l’étude: deux techniciens du laboratoire, opérateurs O1 et O2, ayant une expérience en spermiologie de 8 ans et 21 ans, respectivement; un stagiaire O3, ayant effectué deux mois de stage en spermiologie dans le laboratoire juste avant l’organisation du CQI; et un médecin biologiste O4, formé en spermiologie et participant de façon occasionnelle à l’activité de routine dans le laboratoire.

### Organisation de l’étude

L’étude s’est déroulée dans le laboratoire d’Histologie-Embryologie de la Faculté de Médecine de Sfax. Les prélèvements de spermes appartenaient à des hommes adressés au laboratoire pour exploration d’une infertilité du couple. Les spermogrammes ont été réalisés avec les mêmes microscopes et dans les mêmes conditions pour tous les participants. Trois paramètres spermatiques ont été évalués au cours de ce CQI : la mobilité, la concentration et la morphologie des spermatozoïdes. L’analyse de chaque paramètre spermatique a été effectuée selon les procédures de routine du laboratoire, en se basant sur les recommandations de l’OMS [4]. Plusieurs échantillons ont été analysés au cours de l’étude pour chaque paramètre spermatique. Chaque échantillon a été analysé par tous les participants (variabilité inter-individuelle) et plusieurs aliquotes issues d’un même échantillon ont été testées par chaque participant (variabilité intra-individuelle). Tous les échantillons ont été anonymisés préalablement à l’analyse.

### Préparation des supports

Le sperme a été recueilli au laboratoire par masturbation, après un délai d’abstinence sexuelle de 3 à 5 jours. Le prélèvement a été par la suite placé dans l’étuve à 37°C pendant 30 minutes en moyenne jusqu’à sa liquéfaction.L’évaluation de la mobilité des spermatozoïdes a été faite sur 5 échantillons tripliqués de sperme frais. Afin de faciliter l’analyse des résultats, seule la mobilité totale (progressive et non progressive) [4] a été considérée durant cette étude.Pour l’évaluation de la numération et de la morphologie, les supports ont été préparés à l’avance et stockés afin d’éviter les problèmes de disponibilité des opérateurs et la contrainte de temps.L’évaluation de la numération a été faite sur des aliquotes conservés, issus de neuf prélèvements dupliqués. La préparation de chaque aliquote a été réalisée en ajoutant 100 µl de formaline à 10 % à 1 ml de sperme.La préparation obtenue a été stockée à +4°C jusqu’au moment de l’analyse. Le comptage des spermatozoïdes a été réalisé avec la cellule de Malassez. Cinq échantillons ont servi pour la préparation des lames de morphologie spermatique. Trois frottis sur lame ont été préparés à partir de chaque échantillon. Après coloration au Shorret montage, les lames ont été stockées à température ambiante jusqu’à leur lecture. La classification de David modifiée [11] a été utilisée pour identifier les différentes anomalies morphologiques des spermatozoïdes, et pour déterminer le pourcentage des formes typiques (FT) c.-à-d. de spermatozoïdes ayant unemorphologie normale, duquel nous avons tenu compte au cours de cette étude.

### Etude statistique

La moyenne des lectures des 4 participants a été considérée comme la valeur de référence (valeur vraie) pour tous les paramètres spermatiques étudiés. La variabilité inter-individuelle a été évaluée pour chaque opérateur en calculant le coefficient de variation correspondant pour chaque paramètre spermatique [CV(%)= 100×Écart-type (ET) / moyenne]. De plus, des Courbes de Bland-Altman [12] ont été utilisées pour comparer les différences par rapport à la moyenne (égale à la moyenne des moyennes de lecture des 4 participants) et par rapport aux limites d’acceptabilité supérieure (LAS) et inférieure (LAI). Ces seuils d’acceptabilité correspondent à une différence par rapport à la moyenne ≤15% pour la mobilité et la concentration spermatique, et ≤30% pour la morphologie spermatique, pour tous les échantillons évalués [13, 14]. La variabilité intra-individuelle a été évaluée en calculant le CV pour chaque échantillon dupliqué ou tripliqué, et en le comparant à la moyenne des CV du participant et à la moyenne des CV des 4 participants (valeur référence), et ce pour chacun des paramètres spermatiques analysés.

L’influence du niveau d’expérience sur la variabilité des résultats a été évaluée en déterminant le CV intra- et inter-opérateur dans le groupe G1 (ou groupe d’experts) formé par les deux techniciens O1 et O2; et pour le groupe G2 formé par des opérateurs moins expérimentés (O3 et O4).

## Résultats

### Variabilité inter-individuelle

La moyenne des coefficients de variation inter-participants pour tous les échantillons analysés a été de 12.8% pour la mobilité (allant de 6.8% à 33.5%), 19.8% pour la numération (2.7% à 66.2%), et 48.9% pour la morphologie spermatique (11.7% à 75.9%). Les moyennes des pourcentages de différence par rapport à la moyenne des lectures des 4 opérateurs, ont été déterminées pour chaque participant et pour chaque paramètre étudié (Tableau 1).

**Tableau 1:** variabilité inter-individuelle de la détermination de la mobilité, la numération et la morphologie spermatique par les quatre participants (O1, O2, O3 et O4)

	Mobilité (%)		Numération (×10^6^/ml)		Morphologie (%)	
	Valeur moyenne (± ET)^a^	Moyenne des différences^c^ (%)	Valeur moyenne (± ET)	Moyenne des différences (%)	Valeur moyenne (± ET)	Moyenne des différences (%)
O1	47.6 (2.6)	8.6	19,5 (22.6)	3.2	12.7 (10.7)	13.9
O2	45.6 (8.6)	3.1	19,5 (21.1)	-2.6	11.8 (12.1)	-8.8
O3	42.6 (3.7)	-2.8	21,1 (26.6)	0.3	13.9 (14.3)	26.3
O4	40 (3.2)	-9	21,4 (27.5)	-0.9	5.9 (5.1)	-31.3
Moyenne^b^	44		20.4 (24.4)		11.1	
CV global (%)	12.8		19.8		48.9	
CV groupe G1 (%)	6.34		9.24		39.8^d^	
CV groupe G2 (%)	6.61		13.4		51	

a Moyenne des résultats d’analyse de tous les échantillons.

b Moyenne des valeurs moyennes des quatre participants.

c Moyenne des pourcentages de différence par rapport à la référence (moyenne des 4 participants) pour tous les échantillons.

d p<0.05

**Figure 1:**
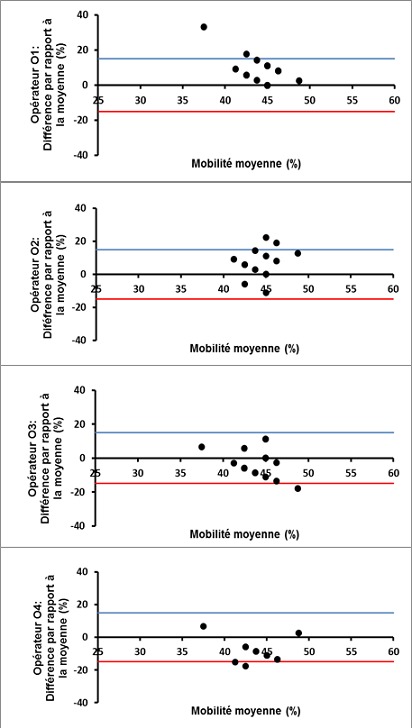
Courbes de Bland-Altman des profils de lecture de la mobilité spermatique par les 4 opérateurs (O1, O2, O3, O4)

**Figure 2:**
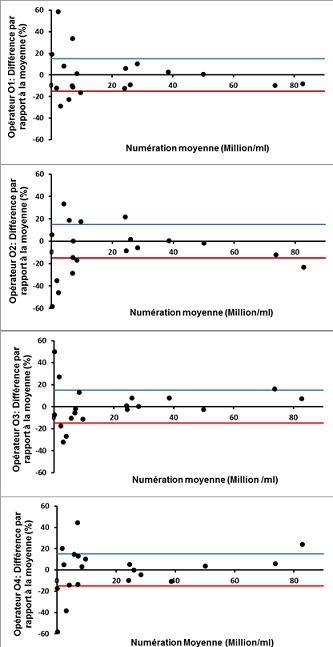
Courbes de Bland-Altman des profils de lecture de la numération spermatique par les 4 opérateurs (O1, O2, O3, O4)

**Figure 3:**
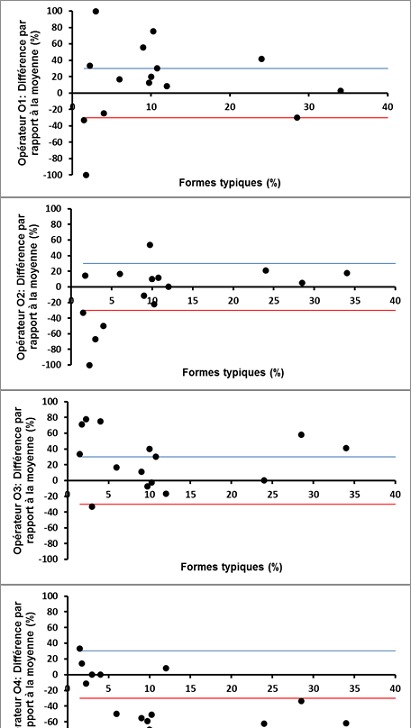
Courbes de Bland-Altman des profils de lecture de la morphologie spermatique (% FT) par les 4 opérateurs (O1, O2, O3, O4)

L’analyse des courbes de Bland-Altman montre que, mis à part quelques erreurs aléatoires de sus ou de sous-estimation, la plupart des lectures des paramètres spermatiques ont été dans les limites d’acceptabilité pour les 4 opérateurs (Figure 1, Figure 2 et Figure 3).

### Variabilité intra-individuelle

**Figure 4:**
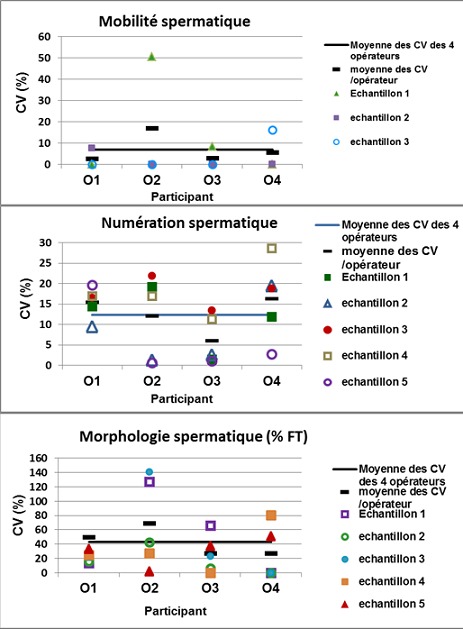
Variabilité intra-individuelle [coefficient de variation, CV(%)] de la détermination de la mobilité, la numération et la morphologie spermatique

Pour chaque participant, un coefficient de variation (CV) moyen a été calculé mesurant le degré de variation intra-individuelle entre les différentes évaluations d’un même échantillon : trois évaluations de chacun des cinq échantillons de sperme pour la mobilité et la morphologie spermatique, et deux évaluations pour chacun des 10 échantillons de sperme pour la numération spermatique.Les valeurs moyennes des CV pour les quatre participants ont été de 6.9% pour la mobilité, 12.3% pour la numération, et 42.7% pour la morphologie spermatique. Pour chaque paramètre spermatique, la distribution des CV relatifs à chaque échantillon par rapport à la moyenne des CV est représentée pour chaque participant dans la Figure 4.

La comparaison des CV intra-opérateur dans les groupes G1 et G2 n’a pas montré de différence significative pour l’évaluation de la mobilité (6.34% vs 6.61%), de la concentration (13.6% vs 11.1%) et de la morphologie spermatique (38.7% vs 26.6%), respectivement (p< 0.05).

## Discussion

La mise en place de programmes d’assurance qualité en biologie de la reproduction est une nécessité. Des contrôles qualité internes (CQI) et externes (CQE) sont nécessaires afin de déceler les erreurs systématiques et aléatoires qui peuvent se mettre en place avec le temps,affectant par conséquence la qualité des résultats [15]. Il est ainsi impératif d’élaborer une démarche d’assurance qualité performante et adaptée pour chaque laboratoire pratiquant des analyses de sperme.

Dans notre travail, nous avons essayé d’élaborer un protocole de CQI simple et reproductible, pouvant servir de modèle pour tout laboratoire de spermiologie, quels que soient son niveau de performance et son débit d’activité.En effet, la préparation et le stockage préalable des échantillons de contrôle de qualité (numération et morphologie spermatique) permettent d’éviter les problèmes de disponibilité des échantillons et des opérateurs et la contrainte de temps, et de minimiser le gaspillage de matériel. Aussi, la possibilité de sélectionner les échantillons nous permet de constituer des séries assez représentatives des échantillons analysés en routine dans le laboratoire. Dans notre étude, les CV intra- et inter-participants pour la mobilités permatique ont été dans les limites d’acceptabilité et similaires aux CV retrouvés dans d’autres études [13]. Malgré la part de subjectivité relativement importante pour ce paramètre et son étroite dépendance du niveau d’entrainement de l’opérateur [16], les CV des groupes 1 et 2 ont été similaires. Ceci souligne l’avantage de l’utilisation du sperme frais au cours du CQ de l’évaluation de la mobilité (comparativement au sperme conservé en paillettes) et de l’homogénéisation des conditions d’analyse (temps, température, microscopes etc.) pour limiter les facteurs de variabilité.

Concernant l’évaluation de la concentration spermatique, le CV inter-participant a été de 19.8%, ce qui est un peu en dehors de la zone d’acceptabilité pour les laboratoires de référence, située aux alentours de 15%. Cet écart peut être expliqué en partie par l’utilisation d’échantillons de sperme conservés, pour lesquels des CV supérieurs à ceux obtenus avec du sperme frais ont été rapportés dans la littérature [17]. De plus, la présence d’opérateurs moins expérimentés et participant de façon irrégulière à l’activité de routine du laboratoire augmenterait d’avantages la variabilité des résultats [18], comme en témoigne l’écart de CV constaté entre G1 et G2 dans notre étude. L’analyse des graphiques de Bland-Altman nous a permis de constater que la variabilité est d’autant plus importante que la concentration de l’échantillon est faible(inférieure à 10 millions/ml dans notre étude), ce qui est en accord avec les constatations d’autres auteurs [16]. Il est en effet recommandé de multiplier et de varier la qualité des échantillons de CQ pour assurer un échantionnage représentatif [19]. La variabilité intra- et inter-participants a été relativement élevée (47.75 % et 48.9 %, respectivement) lors de l´évaluation de la morphologie des spermatozoïdes. L’impact de la différence du niveau d’expérience entre les participants sur la variabilité des résultats a été net. En effet, en considérant les lectures du groupe G1 seulement (techniciens), le CV inter-observateur a été de 39.8%, se trouvant ainsi dans les limites d’acceptabilité établies dans la littérature, allant de 30 à 40% selon les séries, pour la morphologie spermatique [14, 20]. Il a été démontré que le niveau d’expérience influence considérablement les performances des observateurs lors de l’évaluation de la morphologie spermatique [21]. L’expérience de l’observateur est cependant insuffisante pour garantir une moindre variabilité. Le respect des recommandations relatives à l’analyse des anomalies morphologiques, ainsi que la participation régulière à des programmes de formation continue et de CQ sont des conditions nécessaires pour maintenir le niveau de performance et pour minimiser les dérives [15, 20].D’autre part, et comme pour la concentration spermatique, nous avons constaté une variabilité accrue en cas d’altération importante de la morphologie spermatique (Figure 3). En effet, lorsque la valeur cible est faible (proche de zéro), un faible écart des évaluations influence considérablement le CV. Ce pourquoi il est utile de multiplier les procédures d’analyse statistique lors d’un CQ afin de pallier aux limites spécifiques de chaque méthode analytique [4].

## Conclusion

Ce travail nous a aidés à déceler et à évaluer l’ampleur de variabilité de l’évaluation des paramètres spermatiques au sein de notre laboratoire. La simplicité et le faible coût des procédures choisies au cours de ce CQI faciliteront son adoption et sa mise en œuvre de façon régulière dans le laboratoire. Une fois les différences détectées, l’étape suivante est l’identification des sources d’erreurs afin de prendre les actions correctives adaptées.Une formation adéquate et continue du personnel de laboratoire et une participation régulière aux CQ permettrait de minimiser les divergences entre les opérateurs. Cela aurait pour principal avantage d’éviter des répétitions inutiles des demandes d’analyse de sperme, consécutives à des changements de pratiques ou de laboratoires. De ce fait, l’intégration de procédures de CQI dans chaque laboratoire pratiquant des analyses de sperme, et l’organisation régulière de CQE dans une seconde étape, constituent des mesures à entreprendre sans retard afin d’améliorer les performances et la qualité de cette activité.

### Etat des connaissances actuelles sur le sujet

- Malgré les essais de standardisation des procédures d’analyse du sperme, une hétérogénéité des pratiques et des résultats est souvent constatée;
- La mise en place d’outils adéquats pour s’assurer de la fiabilité des résultats d’analyse du sperme dans le laboratoire est une nécessité.

### Contribution de notre étude à la connaissance

- Dans ce travail, nous avons proposé un protocole de contrôle de qualité interne simple et reproductible, pouvant servir de modèle pour tout laboratoire de spermiologie;
- Nos résultats mettent l’accent sur l’importance de la formation du personnel et de l’implantation de programmes de contrôle de qualité dans les laboratoires d’analyse de sperme.
